# Initial Evaluation of the Concept-2 Rowing Ergometer's Accuracy Using a Motorized Test Rig

**DOI:** 10.3389/fspor.2021.801617

**Published:** 2022-01-25

**Authors:** Gunnar Treff, Lennart Mentz, Benjamin Mayer, Kay Winkert, Thomas Engleder, Jürgen M. Steinacker

**Affiliations:** ^1^Division of Sports- and Rehabilitation Medicine, Ulm University, Ulm, Germany; ^2^University Institute of Sports Medicine, Prevention and Rehabilitation, Paracelsus Medical University, Salzburg, Austria; ^3^Institute of Epidemiology and Medical Biometry, Ulm University, Ulm, Germany; ^4^Faculty of Mechatronics and Medical Engineering, University of Applied Sciences, Ulm, Germany

**Keywords:** automated testing, indoor rowing, validity, power output, home-based training

## Abstract

**Introduction:**

The Concept 2 (C2) rowing ergometer is used worldwide for home-based training, official competitions, and performance assessment in sports and science. Previous studies reported a disparate underestimation of mechanical power output positively related to an unclearly defined stroke variability. The aim of this study was to quantify the accuracy of the C2 while controlling for the potentially influencing variables of the rowing stroke by using a test rig for air-braked rowing ergometers and thus excluding biological variability.

**Methods:**

A unique motorized test rig for rowing ergometers was employed. Accuracy was assessed as the difference in mechanical power output between C2 and a reference system during steady (i.e., minimal variations of stroke power within a series of 50 spacemark, no -strokes) and unsteady simulated rowing (i.e., persistent variations during measurement series) while manipulating the stroke variables shape, force, or rate.

**Results:**

During steady simulated rowing, differences between C2 and the reference system ranged 2.9–4.3%. Differences were not significantly affected by stroke shapes (*P* = 0.153), but by stroke rates ranging 22–28 min^−1^ (*P* < 0.001). During unsteady simulated rowing with alterations of stroke force and rate, mean differences of 2.5–3.9% were similar as during steady simulated rowing, but the random error increased up to 18-fold. C2 underestimated mechanical power output of the first five strokes by 10–70%. Their exclusion reduced mean differences to 0.2–1.9%.

**Conclusion:**

Due to the enormous underestimation of the start strokes, the nominal accuracy of the C2 depends on the total number of strokes considered. It ranges 0.2–1.9%, once the flywheel has been sufficiently accelerated. Inaccuracy increases with uneven rowing, but the stroke shape has a marginal impact. Hence, rowers should row as even as possible and prefer higher stroke rates to optimize C2 readings. We recommend external reference systems for scientific and high-performance assessments, especially for short tests designs where the start strokes will have a major impact.

## Introduction

Ergometer training is a common type of training in many outdoor endurance sports, because it provides a highly controllable workout regardless of weather conditions. The SARS-CoV-2 pandemic has furthermore increased the relevance of indoor and home-based training among athletes and recreational athletes and thus of ergometers (Kim et al., 2020; Pedersen et al., 2021). In the sport of rowing, ergometer training is so widely accepted that the World Rowing Federation hosts official world indoor championships on the indoor rowing machine from Concept 2 (C2, Concept 2, Morrisville, VT, USA). The C2 was actually designed as a training device, but is also used by training centers and rowing federations around the world (Smith and Hopkins, 2012) for performance testing to reduce the influence of environmental conditions, which are difficult to control and greatly affect on-water performance (Kleshnev, 2009; Smith and Hopkins, 2011; Malcata and Hopkins, 2014).

The C2 is an air-braked ergometer and resistance is created by a flywheel that becomes accelerated via a handle that is attached to a chain. During each rowing cycle, the rower pulls the handlebar with the coordinated muscle force of legs, trunk and arms, while moving backward on a sliding seat (i.e., drive phase), thereby accelerating the flywheel. Subsequently, the direction of movement is reversed and the rower moves forward to the starting position (i.e., recovery phase). During this phase the flywheel decelerates due to the resistance of the circulated air, but it does not stop immediately. That is due to the rotating mass of the flywheel and the energy stored. This phenomenon is similar to the momentum of an already accelerated rowing boat. It is worth highlighting that the principle of such an air dampened ergometer is substantially different to mechanically braked ergometers, where an external brake controller determines resistance. In an air-damped ergometer such as the C2, it is the rower who determines resistance via stroke force, rate, and length and thereby also the accuracy of the targeted mechanical output.

According to Van Holst (2014), the actual mechanical power output per rowing cycle, usually the key measure of performance (Soper and Hume, 2004), is calculated by the display-computer of the C2, based on measurements of angular velocity (which is used to calculate acceleration and deceleration of the flywheel), the mass of the flywheel, and a constant factor. This approach differs substantially from the physical definition of mechanical power as work per time. Considering the special calculation and the fact that the C2 is employed worldwide for performance measurements of rowers, it is surprising that there is limited and incomplete information about the quality criteria and particularly validity of the C2.

Two validation studies compared the mechanical power output of the C2 vs. a criterion measure (i.e., external force- and displacement-sensors) during rowing. Lormes et al. (1993) postulated a systematic underestimation of approximately 14 W (6.8%) of the C2 (Model C) and Boyas et al. (2006) reported a systematic underestimation of approximately 25 W (7.4%) (Model D). The latter study showed in fact differences in the error's magnitude between novice and trained rowers due to stroke-to-stroke variability. This has been reported by others, too (Smith and Spinks, 1995). Hence, a systematic error is questionable and accurate validation requires the integration of stroke-to-stroke variability.

The stroke-to-stroke variability within a rowing cycle mentioned here may arise from different variables: (i) The shape of the force vs. displacement curves during drive phase may vary due to different anthropometrical portions and/or sequencing of the lower and upper body, resulting in triangular up to rectangular shapes (Kleshnev, 2000). This shape determines where the force peaks during the stroke, i.e., relatively at the front, mid, or end of the stroke. (ii) The ratio of drive (i.e., when the rower pulls and moves backwards) to recovery phase (i.e., when the rower moves forward and does not apply force to the handle) (drive:recovery) varies between rowers. (iii) The consistency of the generated force varies, depending on pacing strategy, ability, and fitness of the rower. (iv) Finally, the consistency of the drive:recovery ratio and/or stroke rate may vary to a lower or higher degree.

However, the unknown impact of these variables and their variability on the calculation of the C2's mechanical power output can almost not be studied in human rowers since these variables cannot be controlled precisely. We therefore developed a unique test rig for rowing ergometers (Mentz et al., 2020), allowing to control all of the aforementioned variables during simulated rowing and providing a robust criterion measure. Using this test rig, it was possible to evaluate the validity of the C2's mechanical power output and to quantify the effects of manipulations of stroke shape, -frequency (via the drive:recovery ratio), -force, and irregularities in these variables. The aim of this study was the first-time quantification of the C2's accuracy while controlling for the potentially influencing variables of the rowing stroke by using a test rig for air-braked rowing ergometers and thus excluding biological variability.

## Materials and Methods

The test setup consisted out of a custom-made test rig for air-braked rowing ergometers and a commercially available C2 rowing ergometer with the manufacturer's PM5 performance monitor (Concept 2, Morrisville, USA). All trials were conducted in a laboratory (18.0–23.1°C, 40–60% relative humidity, 940–967 hPa air pressure). The drag factor, a C2-specific variable that influences the behavior of the flywheel, was set to 145, corresponding to the standard value of the German Rowing Federation (Schwarzrock et al., 2017). This setting was also applied during human ergometer rowing, when those strokes were recorded that are now reproduced by the test bench [see Mentz et al. (2020) for details].

### Test Rig and Criterion Measure

The test rig for air-braked rowing ergometers (see Supplementary Figure 1) has been described elsewhere (Mentz et al., 2020). In short, the rig enables highly reliable rowing strokes [coefficient of variation (CV) < 1%] that are very similar to those of German elite rowers in terms of stroke shape, force, and mechanical power output. The test rig mounts the front part of the C2 without modifying it in any way and a controlled motor moves a sledge that is connected to the ergometer's chain. The chain is equipped with a 100 Hz load cell (U9C, 2 kN, HBM, Darmstadt, Germany) to measure stroke force. A 100 Hz odometer (Limes L120/B1, Kübler, Villingen-Schwenningen, Germany) captures the displacement of the chain. These sensors allow for the exact calculation of mechanical work, which—divided by the duration of the rowing cycle—allows to calculate mechanical power output of the reference system (P_REF_). A custom MATLAB algorithm (Matlab R2018b, The Mathworks, Inc., Natick, MA, USA) was applied to calculate P_REF_.

### Rowing Ergometer

A previously unused C2 indoor rower (Model D, Concept 2, Morrisville, USA) with a PM5 monitor was applied for all tests. Mechanical power output of the C2 (P_C2_) was logged using a third-party app (FLOAT, Ergstick Lmt, Cambridge, UK). According to the manufacturer's information, the FLOAT-App reads the numbers from the PM5 without any manipulation, thereby mirroring the displayed accuracy of 1 W without decimals.

### Test Design

To obtain the main outcome measure, i.e., the difference in mechanical power output between REF and C2 (ΔP_REF−C2_), a series of experiments (specified below) were conducted on the test rig and data for REF and C2 were logged simultaneously. All strokes (i.e., drive phases) applied during the experiments were based on strokes that had originally been recorded during ergometer testing in German national and international elite rowers with an external reference system (Treff et al., 2017, 2018b). These profiles were subsequently implemented via torque control. Due to the principle of torque control, the first strokes are shorter, while force is higher. This is related to the high inertia of the flywheel at the start, when it gets accelerated from stand still. In that situation, maximum torque (which is defined in the input torque control) is reached after a shorter displacement or travel. Of note, this finely mimics behavior and biomechanical limitations of human rowers. For more details regarding the functionality of the test rig please see (Mentz et al., 2020).

### Experiments

**Figure 1:**
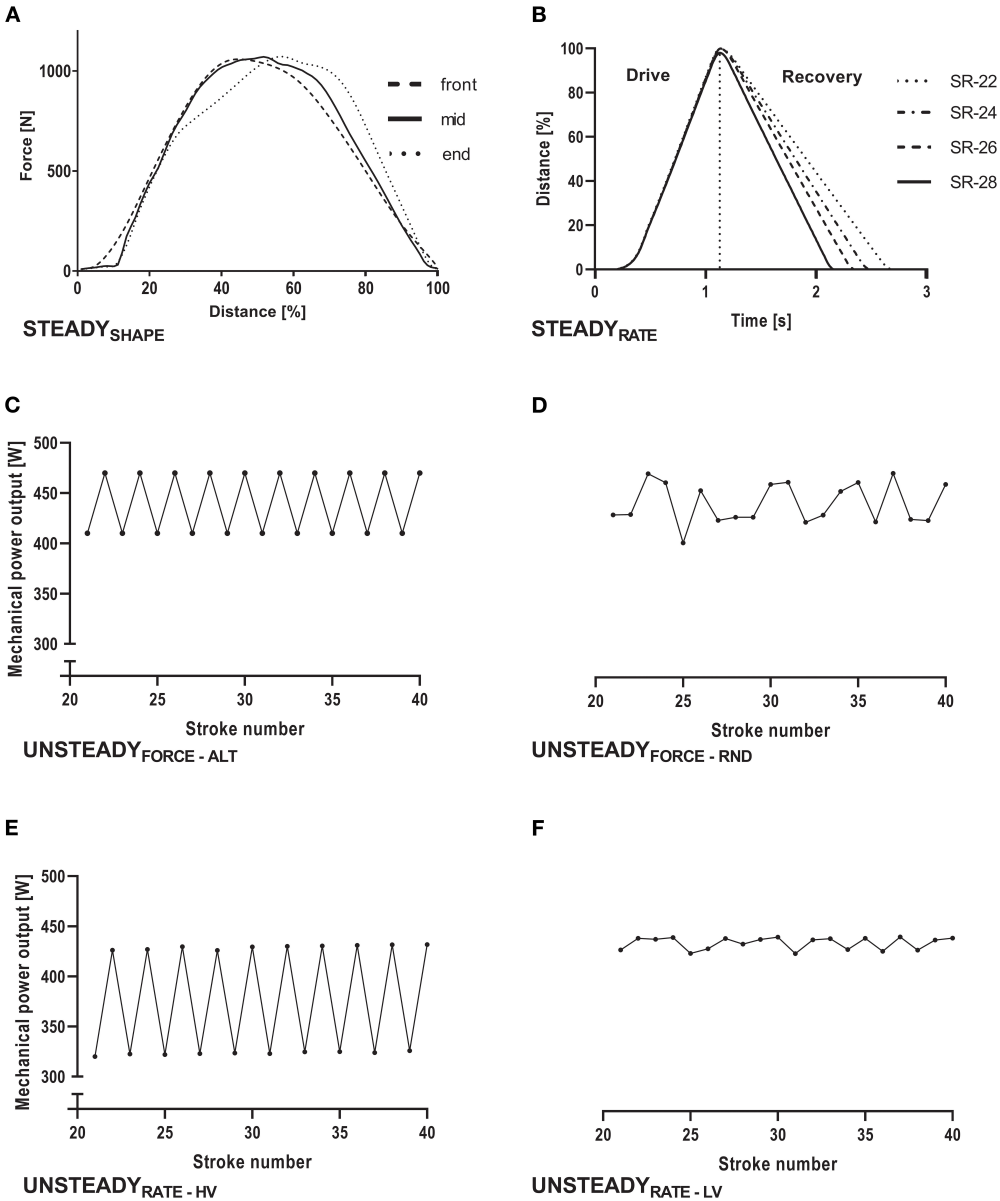
Schematic overview of the experimental design to evaluate differences in mechanical power output between the reference system of a test rig for air-braked rowing ergometers and the Concept 2 Indoor Rower's PM5 Monitor. In **(A,B)** the influence of stroke shape and stroke rate was tested during steady simulated rowing, i.e., stroke-to-stroke variability was as low as possible during each measurement series. **(A)** Shows three different stroke shapes (front-, mid- and end-emphasized) that were applied, while **(B)** visualizes four different stroke rates (SR) of 22, 24, 26 and 28 min^−1^. **(C–F)** depict the mechanical power output (detailed excerpt of strokes 20–40) during unsteady experiments, where stroke-to-stroke variability was experimentally augmented by regular [ALT, **(C)**] or random manipulations of stroke force [RND, **(D)**]. Finally, stroke rate was manipulated highly and regularly [HV, **(E)**] or slightly and randomly [LV, **(F)**], while force was kept constant.

The experiments (Figure 1; Supplementary Table 1) were divided into steady and unsteady simulated rowing. Steady rowing was used to evaluate the impact of (i) stroke shape (STEADY_SHAPE_) and (ii) drive:recovery ratio expressed as stroke rate (STEADY_RATE_) in measurement series with minimum stroke-to-stroke variability. Unsteady rowing was applied to evaluate the impact of persistent fluctuations in (iii) force (UNSTEADY_FORCE_) and (iv) stroke rate (UNSTEADY_RATE_) in measurement series with high stroke-to-stroke variability.

i. **STEADY**_SHAPE_: To evaluate the impact of different force vs. displacement curve shapes on ΔP_REF−C2_, series of 50 strokes each with either a front-, mid-, or end-emphasized profile were completed 10 times. The location of the peak torque relative to the distance was at 45, 51, and 57% of stroke length, respectively (Figure 1A). The stroke rate was set to 27 min^−1^.
ii. **STEADY**_RATE_: To evaluate the impact of different *drive:recovery* ratios on ΔP_REF−C2_, four measurement series where conducted, where the recovery phase lasted 1.6, 1.4, 1.2, or 1.1 s while the drive phase always lasted 1.1 s (Figure 1B). This resulted in drive:recovery rations of 0.69, 0.79, 0.91, and 1.00 and stroke frequencies of 22, 24, 26, and 28 min^−1^, respectively. A mid-emphasized stroke was used for this experiment and each series of 50 strokes was completed twice.
iii. **UNSTEADY**_FORCE_: To evaluate the impact of an unsteady force application on ΔP_REF−C2_, the magnitude of force between strokes within measurement series was modified by alternating the input torque curve of the mid-emphasized profile within two different measurement series, each consisting of 50 strokes completed 10 times. Duration of the recovery phase was kept constant at 1.1 s during each series.

a. Alternating (ALT): The peak-torques of 14.5 or 15.5 Nm were alternated stroke by stroke (Figure 1C).
b. Random (RND): The peak-torques of 14.5 and 15.0 Nm were randomly varied between the strokes (Figure 1D). These peak-torques were chosen to generate a coefficient of variation for stroke-to-stroke variability of 2–5%, corresponding to a stroke-to-stroke variability obtained in human elite rowers (Mentz et al., 2018; Treff et al., 2018a).

iv. **UNSTEADY**_RATE_**:** To assess the effect of permanent changes in the drive:recovery ratio (stroke rate) on ΔP_REF−C2_, the duration of the recovery phases was alternated within three measurement series, while stroke duration and peak torque during the drive phase were clamped. Each series consisted out of 50 mid-emphasized strokes (Figures 1E,F) and was completed 10 times.

a. High variation (HV): The recovery duration of 1.07 s and 1.66 s was alternated stroke by stroke, resulting in stroke rates of 22 and 28 min^−1^ (Figure 1E).
b. Low variation (LV): The recovery durations of 1.2 and 1.3 s were alternated randomly, resulting in stroke rates of 29 and 30 min^−1^, thereby simulating a human variation (Figure 1F).

### Data Analysis and Statistics

The force, displacement, and time data of the test rig were logged using a custom code in Labview 2019 (National Instruments, Texas, Austin, USA) and stroke by stroke mechanical power output was calculated using a custom algorithm in MATLAB (Matlab r2018b, The Mathworks, Inc., Natick, MA, USA). Arithmetic mean, standard deviation, and CV of P_REF_ and P_C2_ were calculated for each trial using SPSS 26 (IBM, Armonk, NY, USA). Accuracy was calculated for each trial as absolute and relative mean difference [100 x ((P_REF_ – P_C2_) / P_REF_)]. ΔP_REF−C2_ between tests was statistically analyzed using a mixed model with fixed effects being *stroke number* (stroke) and *type*. *Type* was defined in STEADY_SHAPE_ (i) as front-, mid-, or end-emphasized stroke shape, in STEADY_RATE_ (ii) as a stroke rate of 22, 24, 26, or 28 min^−1^, and for UNSTEADY_FORCE_ (iii) as alternating (ALT) or random variation (RND) in stroke force within a measurement series; for UNSTEADY_RATE_ (iv) as high or low variation (HV and LV, respectively) in recovery duration within each measurement series. The interaction effect of stroke^*^type was also tested. A graphic visualizing the statistical approach can be found in the supplements (see Supplementary Figure 2). The same model was applied for absolute (i.e., Watt) and relative (i.e., percentage) differences. The mixed model was implemented in SAS (SAS institute, Cary, NC, USA), applying the proc mixed procedure. The level of significance was set to *P* < 0.05 and Bonferroni tests were used for *post-hoc* testing. Effect sizes (partial eta squared η^2^) were considered as small (≥ 0.01 < 0.06), medium (≥ 0.06 < 0.14), or large (≥0.14) (Cohen, 1988).

Due to the previously reported underestimation in mechanical power output of the C2 system during the starting strokes, which in some way was expected to mitigate the impact of the experimental manipulation, we calculated results not only for *all* strokes (i.e., strokes_1−50_) but also exclusively for strokes 6–50 (i.e. exclusion of start strokes; strokes_6−50_). For strokes_6−50_, the same mixed model approach described above was applied.

Due to the impact of the start strokes, these were also excluded for the Bland-Altman plots (Bland and Altman, 1986), in order to analyze bias and limits of agreement (i.e., random error) without the influence of the starting strokes. If ΔP_REF−C2_ indicated a magnitude dependence, the Bland-Altman plots were modified with linear regression analysis and regression based limits of agreement (LoA), as suggested by Bland and Altman (Bland and Altman, 1999).

## Results

### Impact of Shape and Stroke Rate During Steady Rowing

Tables 1, 3 indicate that different shapes did not lead to significant differences of ΔP_REF−C2_ (*P* = 0.153). Differences ranged 12.9–13.9 W (2.9–3.1%) for front, mid, and end emphasized stroke shapes.

**Table 1:** Mean differences in mechanical power output between the reference system of a test rig for air-braked rowing ergometers and the Concept 2 Indoor Rower's PM5 Monitor during steady simulated rowing.

**Data**	**Variable**	**i. STEADY**_**SHAPE**_			**ii. STEADY**_**RATE**_			
		**front**	**mid**	**end**	**SR-22**	**SR-24**	**SR-26**	**SR-28**
	P_REF_, W	432 ± 7	435 ± 4	445 ± 4	329 ± 9	370 ± 7	400 ± 5	450 ± 5
Strokes_1−50_	ΔP_REF−C2_, W	12.9 ± 51.4	13.3 ± 50.5	13.9 ± 51.4	15.1 ± 42.7	15.2 ± 47.4	15.4 ± 49.0	15.6 ± 52.6
	ΔP_REF−C2_, %	2.9 ± 11.1	3.0 ± 11.1	3.1 ± 11.1	4.3 ± 11.2^**^	3.9 ± 11.6^*^	3.8 ± 11.8	3.6 ± 12.3
Strokes_6−50_	ΔP_REF−C2_, W	1.9 ± 1.8	2.3 ± 1.8	2.7 ± 1.8	6.3 ± 1.5	5.2 ± 1.7	4.6 ± 2.0	3.6 ± 2.0
	ΔP_REF−C2_, %	0.5 ± 0.4	0.5 ± 0.5	0.6 ± 0.4	1.9 ± 0.5^++^	1.4 ± 0.5^##^	1.1 ± 0.5	0.8 ± 0.5

*Data are arithmetic mean ± standard deviation. ΔP_REF−C2_: differences in mechanical power output between the reference system of a test rig for air braked rowing ergometers (REF) and the Concept 2 Indoor Rower's PM5 monitor (C2); Strokes_1−50_: all strokes are analyzed; Strokes_6−50_: first five strokes are excluded from analysis; front, mid, and end indicate the location of peak force relative to stroke length; SR: stroke rate (min^−1^);*

** *indicates highly significant difference to SF 24, 26, 28 min^−1^ (P < 0.001);*

* *indicates significant difference to SR 28 min^−1^ (P < 0.05);*

++ *indicates highly significant difference to SR 24, 26, 28 min^−1^ (P < 0.001);*

## *indicates highly significant difference to SR 28 min^−1^ (P < 0.001)*.

**Figure 2:**
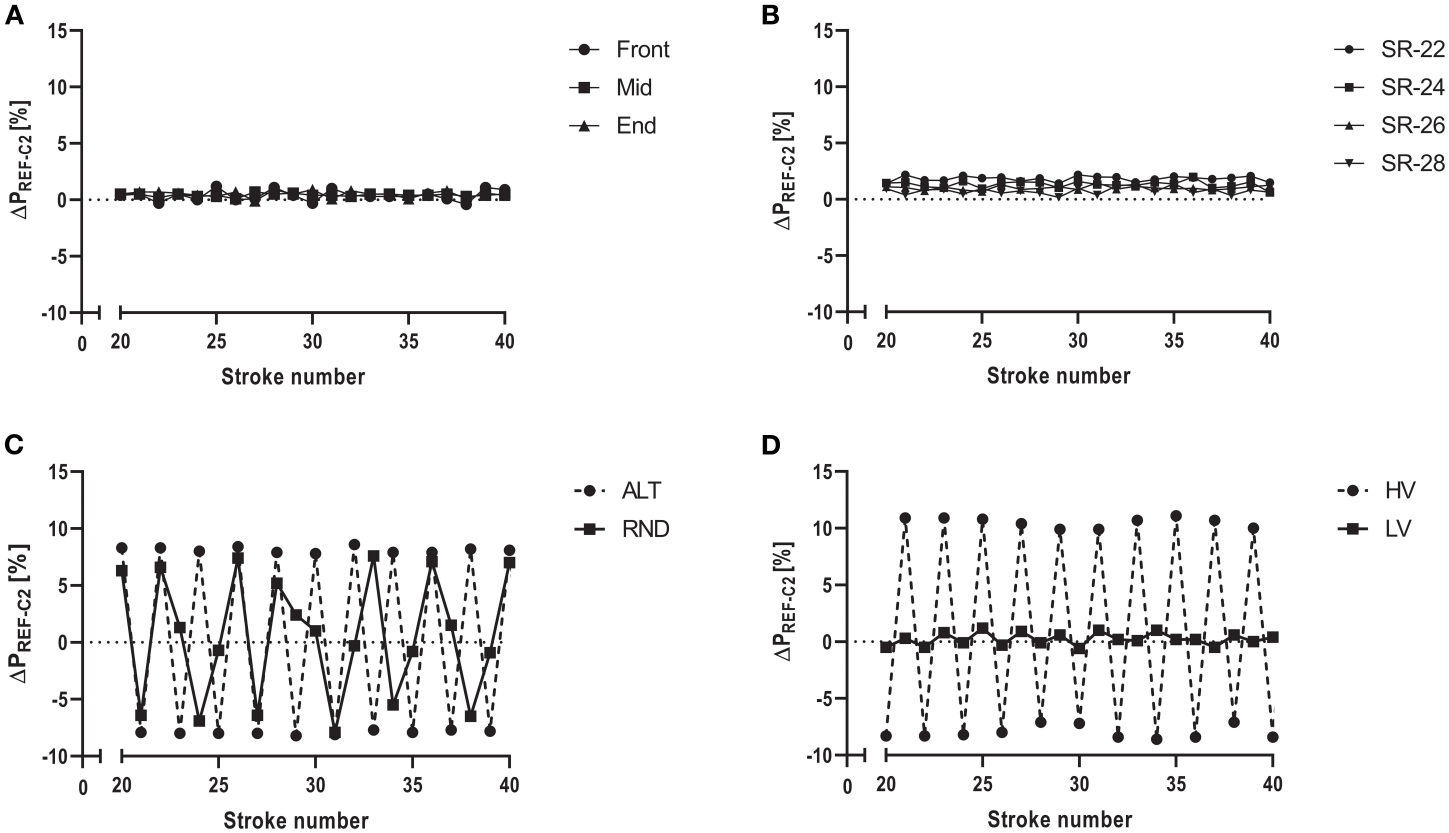
Differences in mechanical power output between the reference system of a test rig for air-braked rowing ergometers and the Concept 2 Indoor Rower's PM5 Monitor (ΔP_REF−C2_ [%]). **(A–D)** show arithmetic mean of 10 repeated measurement series for strokes 20–40 **(A)** Different stroke shapes, i.e., front-, mid- and end-emphasized strokes during steady rowing. **(B)** Four different stroke rates, corresponding to 22, 24, 26, and 28 min^−1^ during steady rowing. **(C)** Persistent variations in stroke force within the measurement series either by regular (ALT) or random (RND) alterations causing unsteady rowing. **(D)** Persistent regular or random variations in stroke rate within measurement series causing either high (HV) or low variations (LV).

On the other hand, differences ranged 15.1–15.6 W (4.3–3.6%) for the four different stroke rates, where a longer duration of the recovery phase for a given drive phase was associated with highly significant differences of ΔP_REF−C2_ (*P* < 0.001). The different drive:recovery ratios (i.e., different stroke frequencies) had a medium or large effect on ΔP_REF−C2_ for strokes_1−50_ and strokes_6−50_. In addition, a large interaction effect was found when all strokes were included. I.e., the stroke rates ranging between 22 and 28 min^−1^ were associated with different ΔP_REF−C2_, noteworthy for absolute and percentage differences (*P* < 0.001). It is worth mentioning that also the analysis without starting strokes was associated with a large (albeit not significant) interaction effect for stroke rate (*P* = 0.23). Accordingly, Figure 2A indicates almost no visible differences between stroke shapes, but Figure 2B clearly shows larger differences to zero and larger differences between strokes caused by manipulations of stroke rate.

### Impact of Force and Stroke Rate Alterations During Non-steady Rowing

The results of the unsteady rowing experiments (iii)–(iv) are shown in Table 2. For continued systematic or random alterations in force generation (i.e., UNSTEADY_FORCE_), the mean differences of the mechanical power output ranged 12.7–13.7 W (2.5–2.6%), i.e., systematic and random alterations had a similar, not significantly different effect on P_REF−C2_. High or low variations of the stroke rate (UNSTEADY_RATE_), in contrast, caused significant differences of 19.8 W (3.9%) or 13.1 W (2.8%). It is worth mentioning that the interaction effects of the experiments (iii)–(iv) (i.e., those employing unsteady simulated rowing) remained highly significant and were accompanied by (very) large effect sizes even when start strokes were excluded.

**Table 2:** Mean differences in mechanical power output between the reference system of a test rig for air-braked rowing ergometers and the Concept 2 Indoor Rower's PM5 Monitor during unsteady simulated rowing.

**Data**	**Variable**	**iii. UNSTEADY**_**FORCE**_		**iv. UNSTEADY**_**RATE**_	
		**a: ALT**	**b: RND**	**a: HV**	**b: LV**
	P_REF_, W	444 ± 31	447 ± 22	380 ± 52	438 ± 10
Strokes_1−50_	ΔP_REF−C2_, W	13.7 ± 58.5	12.7 ± 53.4	19.8 ± 57.6^**^	13.1 ± 52.9
	ΔP_REF−C2_, %	2.5 ± 13.2	2.6 ± 11.9	3.9 ± 15.0^**^	2.8 ± 11.3
Strokes_6−50_	ΔP_REF−C2_, W	3.1 ± 36.2	2.4 ± 22.3	9.8 ± 35.9^**^	1.6 ± 4.4
	ΔP_REF−C2_, %	0.2 ± 8.2^*^	0.3 ± 5	1.3 ± 9.4^**^	0.4 ± 1.0

*Data are arithmetic mean ± standard deviation. ΔP_REF−C2_: differences in mechanical power output between the reference system of a test rig for air braked rowing ergometers (REF) and the Concept 2 Indoor Rower's PM5 monitor (C2); Strokes_1−50_: all strokes are analyzed; Strokes_6−50_: first five strokes are excluded from analysis; ALT and RND indicate regularly and random alternation of stroke force, respectively; HV and LV indicate high regularly and random low alternation of stroke rate, respectively*.

** *indicates highly significant difference to LV (P < 0.001)*.

* *indicates significant difference to RND (P < 0.05)*.

**Table 3:** *P*-values and effect sizes for fixed effects (stroke, type and the interaction of stroke*type) of differences in mechanical power output between the reference system of a test rig for air-braked rowing ergometers and the Concept 2 Indoor Rower's PM5 Monitor within steady and unsteady experiments.

**Effect**	**i. STEADY**_**SHAPE**_		**ii. STEADY**_**RATE**_		**iii. UNSTEADY**_**FORCE**_		**iv. UNSTEADY**_**RATE**_	
	**P**	**η^2^**	**P**	**η^2^**	**P**	**η^2^**	**P**	**η^2^**
Strokes_1−50_	<0.001	0.98	<0.001	0.99	<0.001	0.99	<0.001	0.97
Type_1−50_	0.1534	0.00	<0.001	0.11	0.6331	0.00	<0.001	0.07
Strokes_1−50_*Type	0.6053	0.07	<0.001	0.80	<0.001	0.95	<0.001	0.83
Strokes_6−50_	<0.001	0.16	<0.001	0.53	<0.001	0.97	<0.001	0.84
Type_6−50_	0.0609	0.00	<0.001	0.32	0.0204	0.02	<0.001	0.06
Strokes_6−50_*Type	0.2541	0.08	0.2311	0.46	<0.001	0.97	<0.001	0.84

*Strokes_1−50_ all strokes are analyzed; Strokes_6−50_ first five strokes are excluded from analysis; STEADY_SHAPE_: P-Values and effect sizes for fixed effects between front-, mid- and end-emphasized stroke shapes; STEADY_RATE_: P-Values and effect sizes for fixed effects between stroke rates of 22, 24, 26 and 28 min^−1^. UNSTEADY_FORCE_: P-Values and effect sizes for fixed effects between regularly high and randomly low alternation in stroke force. UNSTEADY_RATE_: P-values and effect sizes for fixed effects between high regularly and low randomly alternation in stroke rate; significance level was set to P < 0.05*.

Figure 2C illustrates details of continuous alterations of force (UNSTEADY_FORCE_), where ΔP_REF−C2_ ranged from −48.2 W (−12.1%) to 49.4 W (10.6%) during ALT and from −39 W (−9.8%) to 43 W (9.1%) during RND. Figure 2D shows details of continuous alterations of stroke rate (UNSTEADY_RATE_), where ΔP_REF−C2_ ranged −25.5 to 45 W (−8 to 11%).

### Start Strokes Largely Affect Overall Accuracy

**Figure 3:**
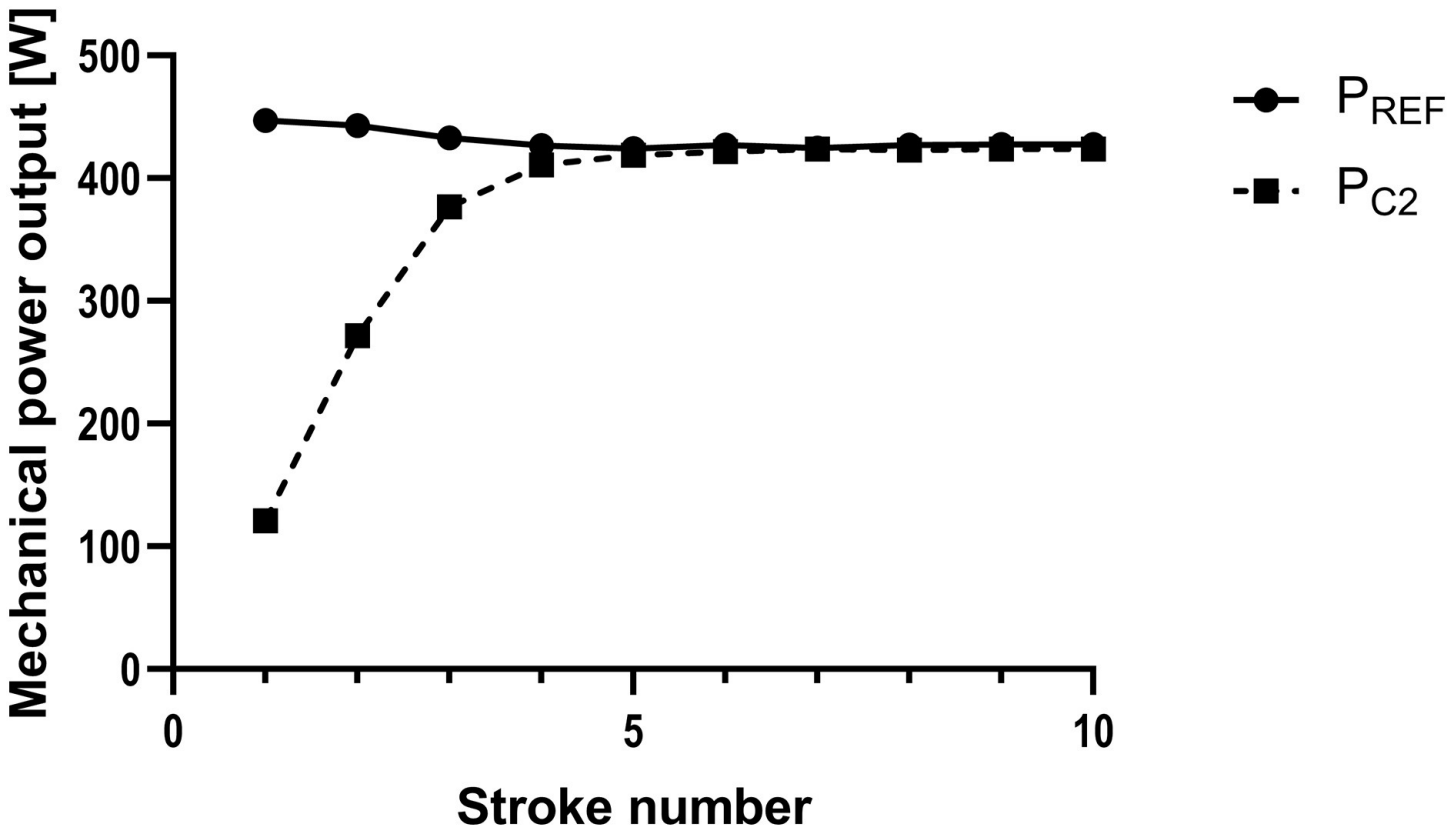
Mechanical power output measured by a reference system of a test rig (P_REF_) and calculated by the Concept 2 Indoor Rower's PM5 Monitor (P_C2_) for the starting strokes (1–10), when the flywheel is accelerated from standstill. Exemplary data from one measurement with steady rowing strokes (using mid-emphasized strokes).

The analysis without starting strokes revealed a substantial reduction of the mean P_REF−C2_ of 81–85% for STEADY_SHAPE_ and 58–71% for STEADY_RATE_, respectively (Figure 3; Table 1). Exclusion of the starting strokes reduced the difference during persistent alterations of force (UNSTEADY_FORCE_) by 77–81% and by 51–86% when stroke rate was persistently altered (UNSTEADY_RATE_) (Table 2).

**Figure 4:**
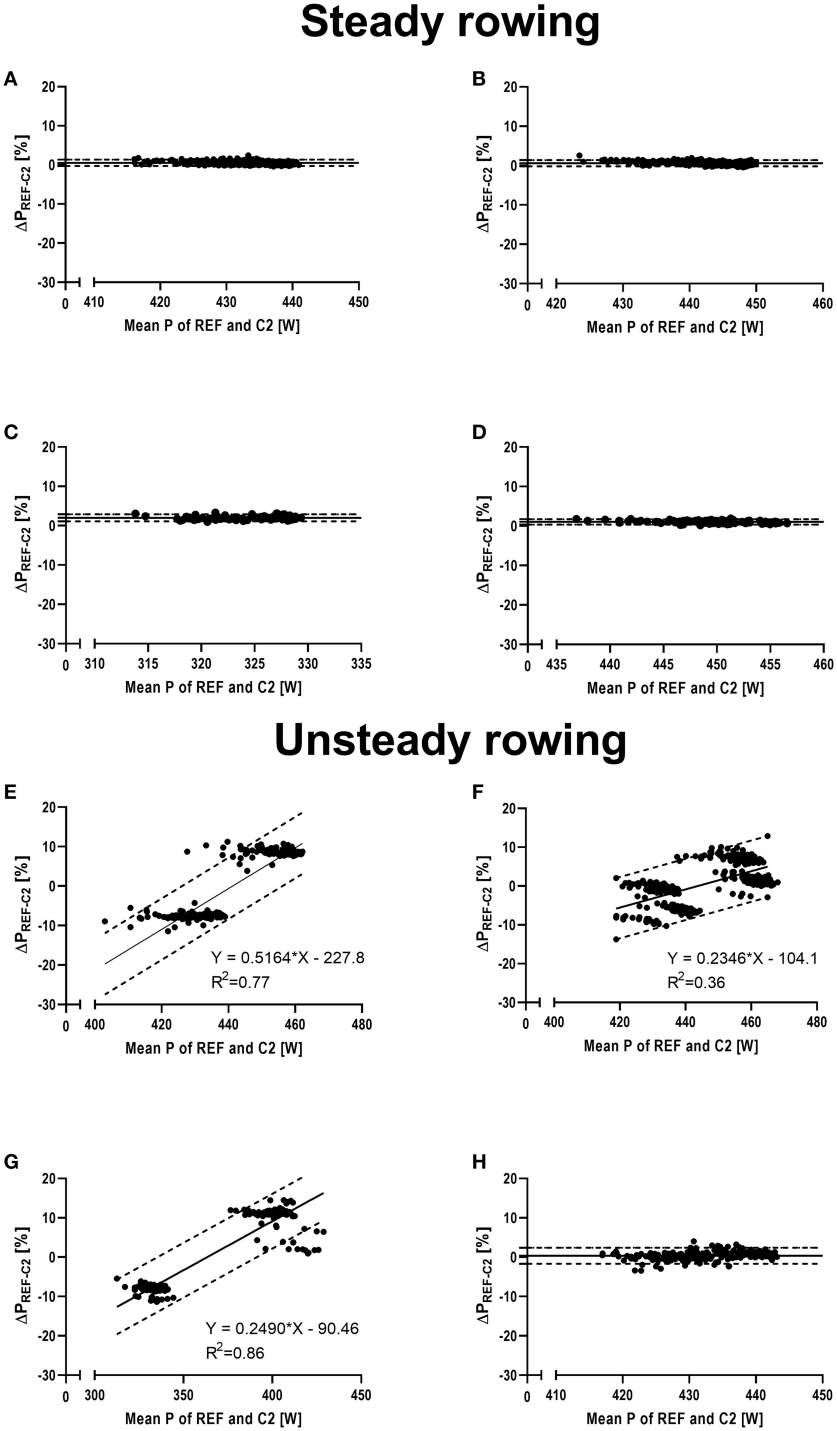
Bland-Altman plots visualizing the percentage differences in mechanical power output between the reference system of a test rig for air-braked rowing ergometers and the Concept 2 Indoor Rower's PM5 Monitor, shown on the y-axis (ΔP [%]). The x-axis shows the mean mechanical power output (P) of both measurement systems. Solid line indicates mean difference and broken dotted lines indicate 95% limits of agreement or, in case of magnitude dependent differences, linear regression analysis and regression-based limits of agreement, respectively. **(A)** Steady rowing with mid-emphasized strokes (mid); **(B)** steady rowing with end-emphasized strokes (end); **(C)** steady rowing with a stroke rate of 22 min^−1^ (SR-22); **(D)** steady rowing with a stroke rate of 28 min^−1^ (SR-28); **(E)** unsteady rowing with high regularly alternating stroke force (ALT); **(F)** unsteady rowing with low randomly alternating stroke force (RND); **(G)** unsteady rowing with high regularly alternations in stroke rate (HV); **(H)** unsteady rowing with low randomly alternations in stroke rate (LV).

The Bland-Altman plots in Figure 4 (please see Supplementary Figure 3 for further details) provide an overview of the experiments with out start strokes, that is the “pure” effect of the manipulations. Systematic bias ranged 0.5–1.9% during steady tests (i–ii) with limits of agreement ranging −0.4–2.9%. The bias during unsteady tests (iii–iv) was mostly magnitude-dependent and limits of agreement ranged −27.5 to 23.3%, depending on the particular experiment and mechanical power output.

## Discussion

The results of this study indicate that the measurement error of the C2 ranges 2.5–4.3% during 50-stroke measurement series, depending on alterations of stroke rate and force, while manipulations of the stroke shape revealed a minor influence. In addition, we found a large underestimation of mechanical power output within the first five strokes when the flywheel was accelerated from standstill. If these strokes were excluded, the error range was reduced to 0.2–1.9%. The random error of measurement increased considerably during unsteady simulated rowing, augmenting the measurement error by up to ~10% for a single stroke. This caused an obvious magnitude dependence of P_REF−C2_.

### Inconsistency Is Linked to Inaccuracy

With very steady rowing strokes within the measurement series (i.e., during STEADY_SHAPE_ and STEADY_RATE_), inaccuracy of the C2 ranged 2.9–3.1% for differently shaped strokes and 3.6–4.3% for different stroke frequencies. Noteworthy, inaccuracy was markedly reduced (up to 6-fold) when ignoring the starting strokes. At the same time, the magnitude of these errors, the narrow limits of agreement as well as their direction (Figure 4) indicate a moderate and systematic error [i.e., consistent bias or offset of the data read from the ergometer (Paton and Hopkins, 2001)] for the stroke shapes, with limits of agreement ranging −0.4–1.4%. The higher errors found for the manipulation of stroke rate reduced gradually (4.3 → 3.6%) from longer to shorter recovery phases, i.e., the closer the drive:recovery ratio approximated 1, the smaller the error became.

During unsteady rowing, the errors from stroke to stroke were markedly amplified (Figures 2C,D; Table 2), but the mean P_REF−C2_ was similar compared to steady rowing tests as long as alterations were not extreme. Noteworthy, the random error [i.e., noise, fluctuations around constant bias (Paton and Hopkins, 2001)] increased considerably in each unsteady rowing experiment, as indicated by the very high standard deviations (Table 2) and considerably wider limits of agreement (Figure 4). In addition, and in contrast to the evenly applied strokes, this error was magnitude dependent. That was the case both with changing stroke force and with large variations in stroke rate (Figures 4E–H).

When the fluctuations in stroke rate increased (i.e., experiment UNSTEADY_RATE_ with high variation) an augmentation of the mean difference from 13.1 to 19.8 W (*P* < 0.001, Table 2) occurred. Hence, the in accuracy of the C2 ergometer is positively associated with stroke to stroke inconsistency. Boyas and colleagues already reported in 2006 a higher accuracy of the C2 in trained rowers (Boyas et al., 2006), who performed with a higher stroke to stroke “consistency” than untrained rowers (Smith and Spinks, 1995). Our results add the information that inconsistency in the drive:recovery ratio has a higher impact in accuracy than variations in stroke force and they also demonstrate a magnitude dependence associated with inconsistency.

The highest degree of inconsistency during all our experiments was observed during the start strokes (Figure 3). Consequently, the exclusion of the first five strokes reduced the mean error substantially, from 2.9–4.3% to 0.5–1.9% during steady simulated rowing experiments and to 0.2–1.3% during unsteady rowing.

Vice versa, the positive association between consistency and accuracy of the power calculation also became evident in the C2's relatively small underestimation of the mechanical power output of only ~13–17 W found in our study during steady simulated rowing. This range is considerably smaller than 14–25 W reported previously in human rowers (Lormes et al., 1993; Boyas et al., 2006). The main reason is likely that our test rig's reliability is much higher than human reliability. The rig has a coefficient of variation of ~0.75% (Mentz et al., 2020), which is much lower (i.e., higher reliability) than variations of 4–5% obtained in elite rowers (Mentz et al., 2018; Treff et al., 2018a) during steady rowing tests. Of note, when the first five strokes were excluded, the mean difference was markedly reduced to 2–7 W, indicating a huge impact of the first five strokes on the mean of a measurements series as long as 50 strokes.

### Underlying Mechanisms

Based on the present data, inaccuracy of the C2 is associated with inconsistency in stroke rate and stroke force. This result is attributable to the measurement principle of the C2 where angular velocity (ω) is the only variable directly measured. This measure also provides the basis for the definition of drive and recovery and therefore each rowing cycle duration. Mechanical power is then calculated by including the so-called drag factor that describes the difference of deceleration and acceleration of the previous rowing cycle and is used as an approximation for the current drive (Van Holst, 2014). This calculation differs substantially from the REF system, which employs a force and displacement sensor to measure all the variables included in the physical definition of mechanical power directly, namely stroke *work* (i.e., *force* times *displacement)* per *time*. In other words, the REF system measures the mechanical power applied to the chain by the rower, whereas the C2 measures the impact of the rower's power output on the behavior of the flywheel. It is noteworthy that both approaches ignore the work of the rower during the recovery phase, when no force is applied to the handle and thus no acceleration of the flywheel occurs (Lindenthaler et al., 2018).

The particularly extreme behavior of the flywheel during the starting strokes allows to gain deeper understanding of our results: Since there is no previous recovery period and therefore no drag factor at stroke #1, the PM5 assumes a default value, which likely contributes to the relatively high error. Furthermore, the first few strokes are different from the subsequent ones, because the flywheel has to be accelerated from standstill. Such acceleration of the mass requires a relatively high amount of energy and a considerable amount of this energy is stored in the flywheel, which therefore continues to rotate even if the rower does not pull the handle while moving himself forward on the ergometer. Of note, this energy is not captured by the PM5. Finally, the difference between acceleration and deceleration is higher the first strokes than between subsequent ones. This causes the substantial underestimation of P_C2_ during the start (personal communication with Peter Dreissigacker, CEO Concept 2) and also supports the results of our study and especially the higher differences observed during unsteady rowing.

The stroke shape does obviously not influence the ratio of acceleration and deceleration substantially (STEADY_SHAPE_ Table 1; Figures 2A, 4A,B) and therefore the effect of the different shapes on ΔP_REF−C2_ is small. But, similar to the start strokes, the C2 underestimates mechanical power output when the deceleration is high relative to the acceleration, which is the case when recovery phases are relatively long during steady rowing (STEADY_RATE_), This causes a strong decrease of rotational velocity during the recovery phase. Consequently, ΔP_REF−C2_ is high when the drive:recovery ratio is low (e.g., 0.69, stroke rate 22 min^−1^) and decreases when the ratio approaches 1 (stroke rate 28 min^−1^). The same effect and additional fluctuations of the flywheel's deceleration and acceleration between consecutive strokes during unsteady rowing in experiments iii and iv demonstrate an extreme miscalculation (Figures 2C,D) on a stroke to stroke level, which, however, almost balances out on average.

It is noteworthy, that the C2 aims to mirror the relationship between mechanical power output and boat speed (personal communication with Peter Dreissigacker, CEO Concept 2). At the start of a race the rower generates substantial power to accelerate the boat from standstill, but the boat speed is relatively low, because of the inertia due to the boat's and the rower's mass and due to the resistance of the water. The same occurs in the C2 that directly links power to pace and therefore estimates a low speed as a consequence of the huge underestimation of the first strokes. So, in this context, a weakness turns out to be a strength, because if mechanical power output was calculated “correctly” (i.e., *work* per *time*), the high mechanical power output at the start would result in an implausible high speed.

### Practical Implications

Our results suggest that rowing as evenly as possible in regard to stroke force and stroke rate will result in less underestimation and thus “better” results on the C2 for a given mechanical power output. This is the case when rowing with high stroke rates and might partly explain—beside dominating biomechanical and physiological reasons—why many rowers prefer high stroke rates in ergometer competitions, at least according to our own observations. In addition, a high number of strokes also ensures that the underestimation of the starting strokes becomes less influential.

On the other hand, when rowing with low stroke rates during training or testing for basic endurance performance like the 6-km test, different drive:recovery ratios will clearly contribute to differences between or within rowers to a small degree. Based on our results and even when excluding the start strokes, the mean difference of 1.9% declines to 1.4% due to a slight alteration in drive:recovery corresponding to a stroke rate of 22 or 24 · min^−1^ for a given drive phase (Table 1). Consequently, a pace of e.g., 1:45.0 min 500 m^−1^ (302.3 W) will increase to 1:45.2 min 500 m^−1^ (300.8 W) cumulating to a difference of 2.4 s during over a virtual 6,000 m distance (i.e., from 21:00.0 min to 21:02.4 min)—notably for the identical drive phase. When testing performance at low stroke rates, we therefore recommend to keep drag factor and stroke rate fixed between tests and between rowers. However, the modification of the C2 with an external reference system would be ideal (Treff et al., 2018b).

Due to the huge underestimation of the first strokes such a reference system seems to be indispensable when aiming to capture start strokes or conducting tests of short duration (e.g., 20-s all out testing). Noteworthy, it is likely that such tests will become more relevant with the possible shortening of the race distance to 1,500 m at the 2028 Olympic Games. Likewise, such a reference system is appropriate whenever the recording of physically exact performance is necessary in scientific contexts. Finally, we recommend paying close attention to always stopping the flywheel before ergometer performance tests to avoid further inaccuracies.

### Limitations

Our study has some limitations worth mentioning. First of all, even though the test rig produces very reliable rowing strokes (CV < 1%), there is still some variability left influencing the results to some extent. In addition, we were not able to generate high stroke frequencies, thereby limiting the transferability of our results for frequencies above ~33 min^−1^ actually applied in ergometer racing. Finally, we conducted all testing on the same ergometer and it remains unclear to which extent our results can be transferred to other C2 ergometers. This is an area for future research just like the evaluation of other types of ergometers that become increasingly popular.

## Conclusion

The error of the C2 ranges 2.5–4.3% if all strokes of a 50-stroke series are included, but it is considerably reduced to 0.2–1.9% once the flywheel has been sufficiently accelerated. Uneven rowing is the main reason for increased inaccuracy. Hence, rowers should row as even as possible and prefer higher stroke rates to minimize underestimation of their performance. Since there is currently no option to exclude the enormously underestimated start strokes, the nominal accuracy of the C2 depends on the total number of strokes considered. We recommend to apply external reference systems for scientific and high-performance assessments of rowers, especially for short tests designs where the start strokes have a major impact.

## Data Availability Statement

The raw data supporting the conclusions of this article will be made available by the authors, without undue reservation.

## Author Contributions

GT developed the research question and study design, conducted data analysis and interpretation, drafted and revised the manuscript. LM developed the study design, collected the data, analyzed and interpreted the data, drafted and revised the manuscript. BM designed, conducted, and interpreted the statistical analysis. KW analyzed data and reviewed the manuscript. TE developed the research question and study design and supported data collection. JS analyzed and interpreted the data and reviewed the manuscript. All authors have read, approved the final version of the manuscript, and agree with the order of presentation of the authors.

## Conflict of Interest

The authors declare that the research was conducted in the absence of any commercial or financial relationships that could be construed as a potential conflict of interest.

## Publisher's Note

All claims expressed in this article are solely those of the authors and do not necessarily represent those of their affiliated organizations, or those of the publisher, the editors and the reviewers. Any product that may be evaluated in this article, or claim that may be made by its manufacturer, is not guaranteed or endorsed by the publisher.
