# Supplementary material for: Initial Evaluation of the Concept-2 Rowing Ergometer's Accuracy Using a Motorized Test Rig
Source: Front Sports Act Living. 2022 Jan 25;3:801617. doi: 10.3389/fspor.2021.801617 (PMC8821892; doi:10.3389/fspor.2021.801617)
Supplement: Supplementary file 1 [file Table_1.docx]

Supplementary Table 1 Experimental design to evaluate differences in mechanical power output between the reference system of a test rig for air-braked rowing ergometers and the Concept 2 Indoor Rower’s PM5 Monitor.

| **Experiment** |  | **Steady rowing (minimum stroke-to-stroke variability)** | | | | | | | |  | **Unsteady rowing (substantial stroke-to-stroke variability)** | | | | |
| --- | --- | --- | --- | --- | --- | --- | --- | --- | --- | --- | --- | --- | --- | --- | --- |
| **Subgroup** |  | **i. shape** | | |  | **ii. stroke rate** | | | |  | **iii. force** | |  | **iv. stroke rate** | |
| **Measurement series** |  | **front** | **mid** | **end** |  | **SR-22** | **SR-24** | **SR-26** | **SR-28** |  | **ALT** | **RND** |  | **HV** | **LV** |
| **Drive** | **Stroke shape** | Front | Mid | End |  | Mid | Mid | Mid | Mid |  | Mid | Mid |  | Mid | Mid |
|  | **Peak to length, %** | 45 | 51 | 56 |  | 51 | 51 | 51 | 51 |  | 51 | 51 |  | 51 | 51 |
|  | **Peak force, N** | 1062 | 1067 | 1073 |  | 1126 | 1125 | 1124 | 1123 |  | 1056-1144 | 1061-1115 |  | 1108 | 1167 |
|  | **Variation** | CON | CON | CON |  | CON | CON | CON | CON |  | ALT | RND |  | CON | CON |
| **Recovery** | **Duration, s** | 1.2 | 1.2 | 1.2 |  | 1.6 | 1.4 | 1.2 | 1.1 |  | 1.1 | 1.1 |  | 1.07/1.66 | 1.20/1.30 |
|  | **Variation** | CON | CON | CON |  | CON | CON | CON | CON |  | CON | CON |  | ALT | RND |
| **Stroke rate, • min-1** | | 27 | 27 | 27 |  | 22 | 24 | 26 | 28 |  | 29 | 29 |  | 22/28 | 29/30 |
| **CV, %** |  | 1.5 | 1.0 | 0.9 |  | 2.6 | 2.0 | 1.6 | 1.0 |  | 7.0 | 5.0 |  | 13.7 | 2.1 |

Stroke shape: front-, mid- or end-emphasized relative to location of peak force; Peak to length: location of peak force relative to stroke length; Variation: CON, variable kept constant within each measurement series; ALT: variable was regularly alternated stroke by stroke within measurement series; RND: variable was alternated randomly within each measurement series; CV: Coefficient of variation.
