# Supplementary material for: Initial Evaluation of the Concept-2 Rowing Ergometer's Accuracy Using a Motorized Test Rig
Source: Front Sports Act Living. 2022 Jan 25;3:801617. doi: 10.3389/fspor.2021.801617 (PMC8821892; doi:10.3389/fspor.2021.801617)
Supplement: Supplementary file 2 [file Data_Sheet_1.docx]

**Supplementary Figure 1**Custom test rig as described by Mentz et al. for air-braked rowing ergometers. Here shown with a Concept 2 indoor rower (Model D, Concept 2, Morrisville, USA).

**Supplementary Figure 2**Illustration of the statistical approach used to analyze the differences in mechanical power output (ΔP) between the reference system of a test rig (REF) for air-braked rowing ergometer and the Concept 2 Indoor Rower’s PM5 Monitor (C2) when manipulating stroke shape, rate, or force during steady and unsteady simulated rowing. The effects aimed to analyze are shown in blue. Fixed effects of the mixed model were the number of 50 consecutive strokes (i.e., stroke_1-50_) or of 45 strokes (i.e., stroke_6-50_), type, and stroke*type. *TYPE* was: STEADY: steady simulated rowing in regard to stroke-by-stroke variability; UNSTEADY: unsteady simulated rowing in regard to stroke-by-stroke variability; SHAPE: different shapes of force-displacement curves; RATE: different stroke rates due to different *drive:recovery* ratios where the duration of the drive was clamped and duration of recovery was manipulated; FORCE: different stroke force; front: front-emphasized stroke shape; mid: mid-emphasized stroke shape; end: end-emphasized stroke shape; SR-22: stroke rate of 22 •min^-1^; SR-24: stroke rate of 24 •min^-1^; SR-26: stroke rate of 26 •min^-1^; SR-28: stroke rate of 28 •min^-1^; ALT: alternating variation in stroke force; RND: random variation of stroke force; HV: high variation in recovery duration; LV: Low variation in recovery duration. See text and supplemental Table S1 for details.

**Supplementary Figure**
Three Bland-Altman plots visualizing the percentage differences in mechanical power output between the reference system of a test rig for air-braked rowing ergometers and the Concept 2 Indoor Rower’s PM5 monitor on the y-axis (ΔP [%]). The x- axis shows the mean of mechanical power output (P) of both measurement systems. Solid line indicates mean difference and broken dotted lines indicate 95% limits of agreement. A: steady rowing with front-emphasized strokes (front); B: steady rowing with a stroke rate of 24 •min^-1^ (SR-24); C: steady rowing with a stroke rate of 26 •min^-1^ (SR-26).
