# Supplementary figures and images for: Initial Evaluation of the Concept-2 Rowing Ergometer's Accuracy Using a Motorized Test Rig
Source: Front Sports Act Living. 2022 Jan 25;3:801617. doi: 10.3389/fspor.2021.801617 (PMC8821892; doi:10.3389/fspor.2021.801617)

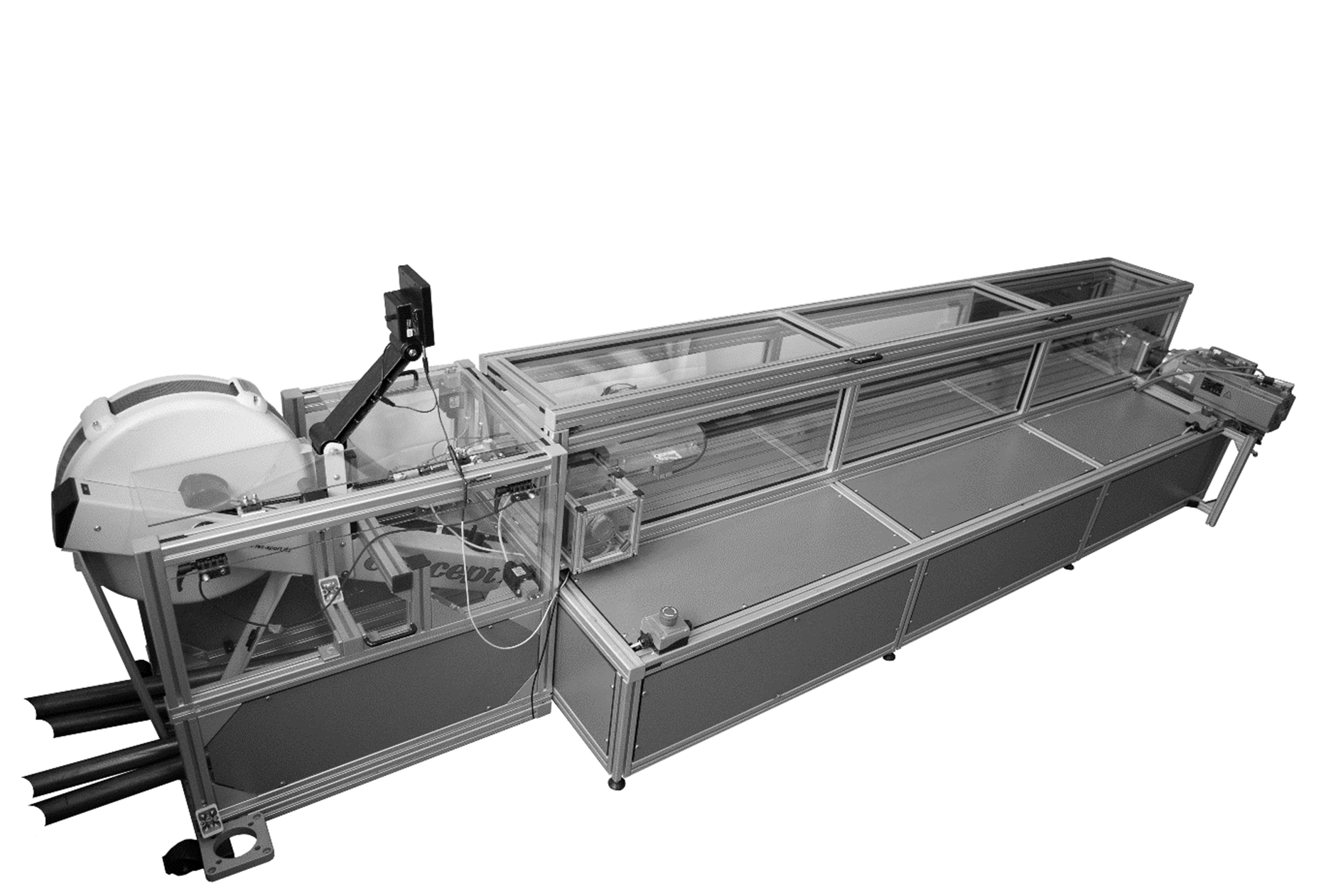

Supplement: Supplementary file 3 [file Image_1.TIF]

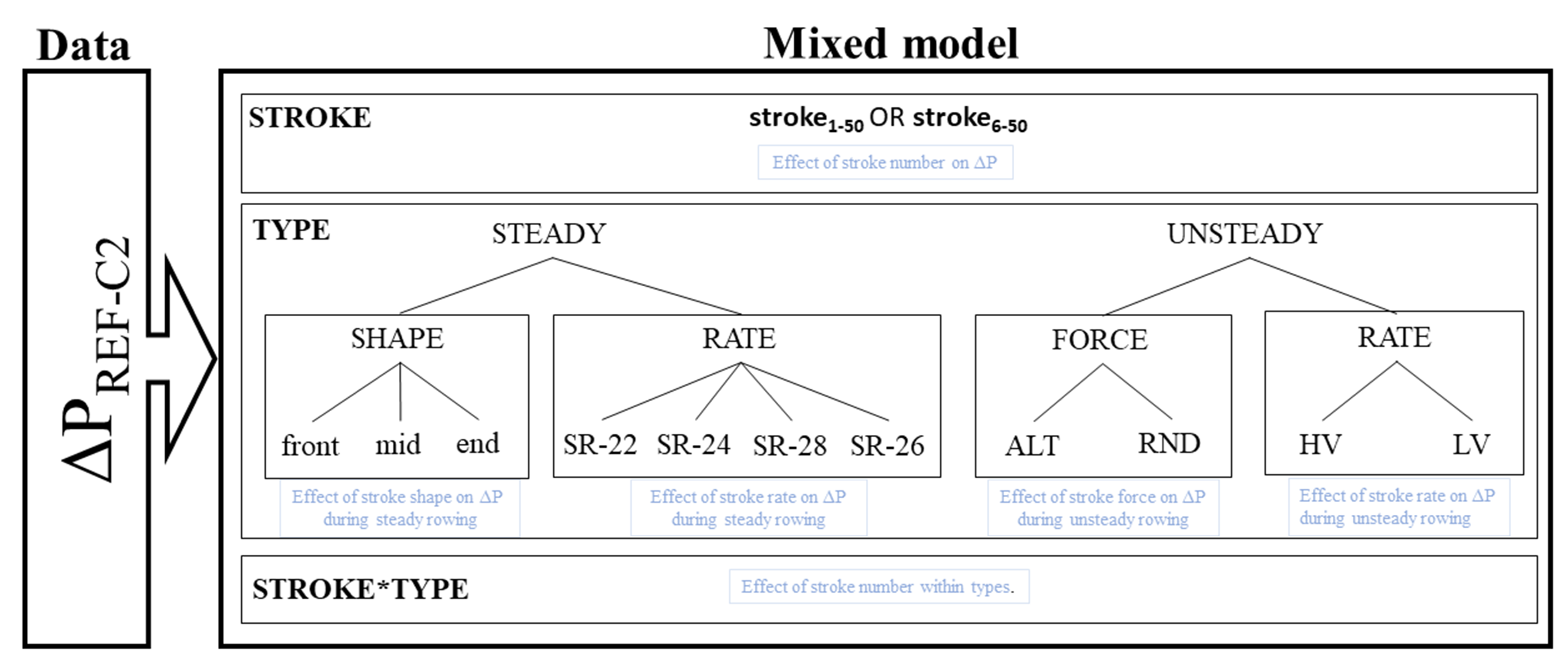

Supplement: Supplementary file 4 [file Image_2.TIF]

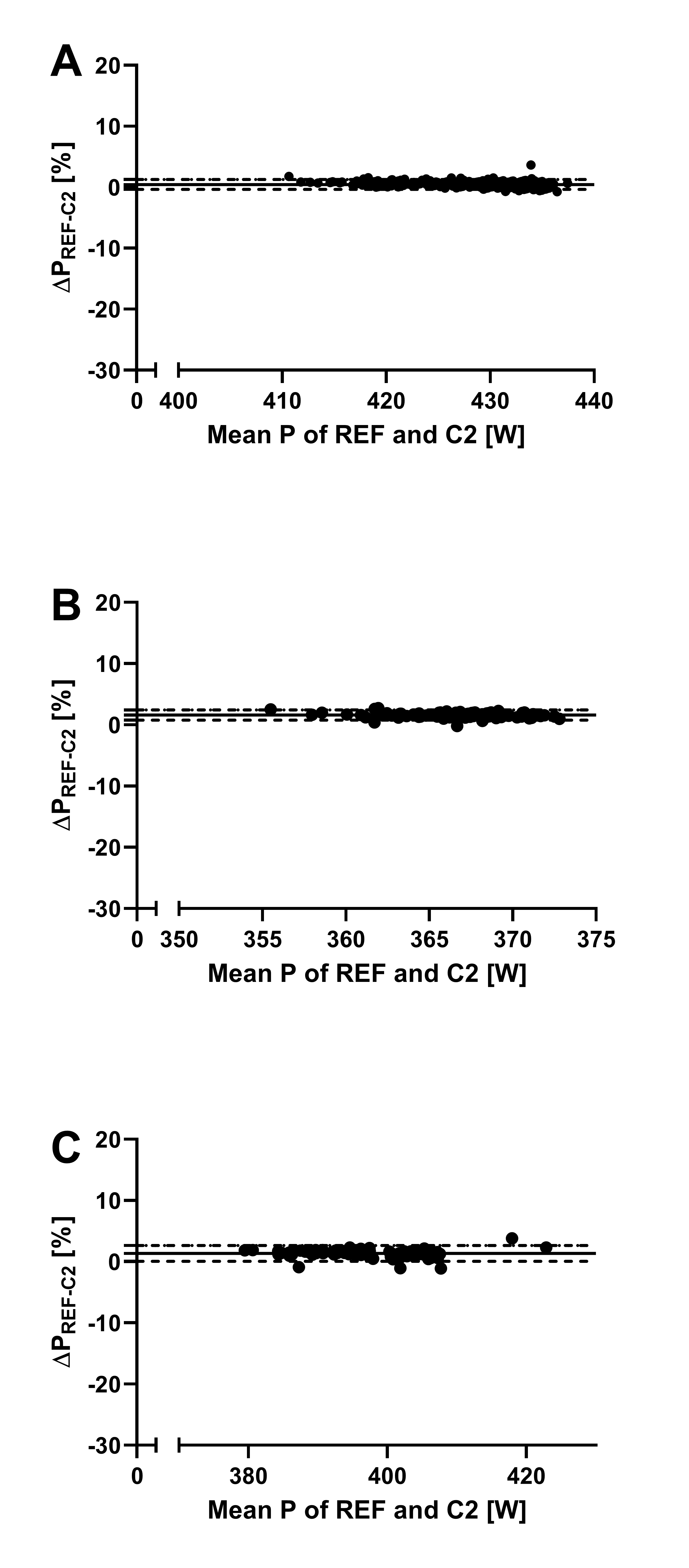

Supplement: Supplementary file 5 [file Image_3.tif]
